# Master equation modeling of water dissociation in small ionic water clusters: Ag^+^(H_2_O)_*n*_, *n* = 4–6†

**DOI:** 10.1039/d4ra03518f

**Published:** 2024-07-12

**Authors:** Michael Hütter, Gabriel Schöpfer, Magdalena Salzburger, Martin K. Beyer, Milan Ončák

**Affiliations:** a Institut für Ionenphysik und Angewandte Physik, Universität Innsbruck Technikerstraße 25 6020 Innsbruck Austria Martin.Beyer@uibk.ac.at Milan.Oncak@uibk.ac.at

## Abstract

We model temperature-dependent blackbody infrared radiative dissociation (BIRD) rate coefficients of Ag^+^(H_2_O)_*n*_, *n* = 4–6, a system with loosely bound water molecules. We employ a master equation modeling (MEM) approach with consideration of absorption and emission of blackbody radiation, comparing single and multiple-well descriptions. The unimolecular dissociation rate coefficients are obtained using the Rice–Ramsperger–Kassel–Marcus (RRKM) theory, employing two approaches to model the sum of states in the transition state, the rigid activated complex (RAC) and the phase space limit (PSL) approach. A genetic algorithm is used to find structures of low-lying isomers for the kinetic modeling. We show that the multiple-well MEM approach with PSL RRKM in the *All Wells and Transition States Are Relevant* (AWATAR) variant provides a reliable description of Ag^+^(H_2_O)_*n*_ BIRD, in agreement with previously published experimental data. Higher-lying isomers contribute significantly to the overall dissociation rate coefficient, underlying the importance of the multiple-well ansatz in which all isomers are treated on the same footing.

## Abstract 2

Master equation modelling within the AWATAR ansatz reveals dissociation kinetics in hydrated silver cations, Ag^+^(H_2_O)_*n*_, *n* = 4–6.

## Graphical abstract

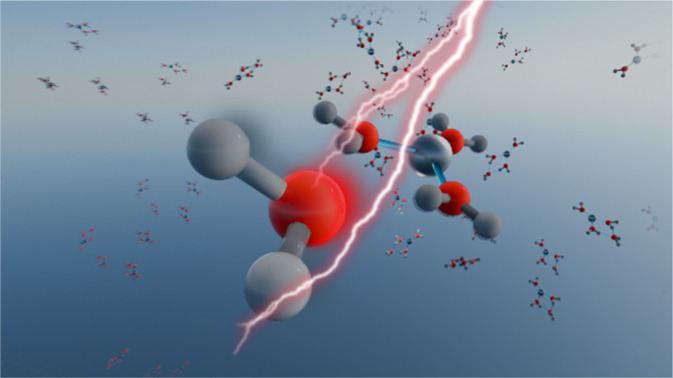

## Introduction

Charged molecular clusters serve as model systems for ion solvation.^1^ Among them, hydrated metal ions hold a prominent place.^2–6^ Delving into their gas-phase properties can substantially enhance our understanding of metal–ligand interactions, like ligand binding energies,^7–9^ the onset of hydrogen bonding,^10–12^ or photochemical hydrogen evolution.^13^ In the gas phase, the analog of silver chloride precipitation can be observed by mass spectrometry,^14^ and static calculations^15,16^ as well as *ab initio* molecular dynamics simulations illustrating the formation of single-molecule precipitates in finite water clusters.^17^

Binding energies for Ag^+^(H_2_O)_*n*_, *n* = 1–6, were previously measured by Holland and Castleman using high-pressure mass spectrometry.^18^ The investigations revealed that the initial two water molecules bind significantly stronger than the subsequent ones. This phenomenon is attributed to the hybridization of the 5 s and 4d orbitals of Ag^+^, resulting in a coordination number of two. Complementing this, bond energies were measured by Armentrout *et al.* through collision-induced dissociation (CID) experiments on Ag^+^(H_2_O)_*n*_, *n* = 1–4.^19^

As an important model system for silver coordination chemistry, Ag^+^(H_2_O)_*n*_ has been studied repeatedly by quantum chemistry,^20–29^ starting with calculations of Ag^+^(H_2_O) by Chattaraj and Schleyer,^20^ and more recently also served as a test case for four-component relativistic density functional theory (DFT) calculations.^29^ Feller *et al.* obtained a wide variety of structures for *n* = 3, 4, and identified the (3 + 0) and (3 + 1) structures as global minima for *n* = 3 and *n* = 4, respectively, using wave function based methods.^21^ Here, (*n* + *m*) denotes a structure with *n* water molecules in the first and *m* in the second solvation shell. DFT calculations by Fox *et al.* predicted linear two-fold coordination for both *n* = 3 and *n* = 4, with hydrogen bonding starting at *n* = 3.^23^ Infrared multiple photon dissociation (IRMPD) spectroscopy of Ag^+^(H_2_O)_*n*_, *n* ≤ 4, by Ohashi and co-workers revealed a three-coordinated structure for Ag^+^(H_2_O)_3_Ar,^28^ while hydrogen bonding was observed in spectra of Ag^+^(H_2_O)_3_.^25^ The authors interpreted this with a (3 + 0) structure as the global minimum, which partly converts to a (2 + 1) structure at the finite temperatures of a molecular beam experiment. Ag^+^(H_2_O)_4_ features (3 + 1) and (2 + 2) structures, the latter in linear two-fold coordination with two water molecules hydrogen bonded in the second solvation shell.^25^ X-ray experiments by Persson and Nilsson in aqueous solution indicate a coordination number of four in a linearly distorted tetrahedral arrangement.^30^ Busato *et al.* pointed out that additional water molecules may be present at only slightly larger distance.^31^ Theory and experiment are thus in accordance that the coordination chemistry of aqueous Ag^+^ is very complex, and a large number of isomers must be expected with relatively small energy differences.

In this study, we use a complementary approach for the determination of water binding energies in the hydration shell of Ag^+^ in the gas phase. Unimolecular rate coefficients of blackbody infrared radiative dissociation (BIRD)^32–39^ at different temperatures for mass-selected Ag^+^(H_2_O)_*n*_, *n* = 4–6, have been published previously.^40^ We employ master equation modeling (MEM) to compute temperature-dependent BIRD rate coefficients. These computational results are then compared with experimental rate coefficients.^40^ MEM parameters are adjusted to match the experimental results, thereby obtaining activation energies for water evaporation. Traditionally, MEM rate coefficient calculations predominantly focus on the isomer with the lowest energy.^41,42^ However, we aim here for a more realistic description of the accessible phase space of the system by using a recently developed multiple-well MEM method^43,44^ to determine the binding energies of Ag^+^(H_2_O)_*n*_, *n* = 4–6. The configurational space of Ag^+^(H_2_O)_*n*_ is explored with a genetic algorithm.

## Methods

### Genetic algorithms

Our own implementation of a genetic algorithm (GA) was employed to identify energetically low-lying isomers.^45^ Generally, a GA is based on a higher-level heuristic, inspired by Darwin's process of natural selection. A finite initial population *P*_0_, consisting of *n* members, is chosen randomly from the search space *Ω*. This initial population is then iteratively translated to new populations by applying a map *τ*, known as transition rules, to the previous population with *P*_*i*+1_ = *τ*(*P*_*i*_) until a termination criterion or a maximum number of *i* = *i*_max_ cycles is met.

Here, we set the maximum number of cycles and population size to 10. Further, a real-valued genetic representation of the candidate solution is employed, allowing for direct genotype-phenotype mapping and thus an easy implementation of genetic operators.^46^ Namely, once the number of atoms and atom types are chosen, different isomers are represented by specific atom positions, given in Cartesian coordinates. However, to initialize valid phenotypes, a limitation of the individual atom positions to a subset *M* ⊆ *Ω*, with *M* = *X* × *Y* × *Z*, and a minimum interatomic distance chosen to be (8 × 8 × 8) Å^3^ and 0.6 Å, respectively, is implemented.

Further, *τ* was chosen to combine selection, crossover, and mutation functions. For selection, a proportional selection function was implemented, meaning that given an objective function, population members are chosen in proportion to their fitness. The fitness function simply corresponds to the electronic energy *E* which was evaluated using Gaussian 16 (ref. 47) after a local optimization of the structures, employing the BLYP/def2SVP level of theory. This additional localized gradient-driven minimization results in a significantly improved efficiency of the algorithm as local structures can be resolved much better using gradients.

In the crossover process, two parent molecules are chosen at random. Then, a one-point crossover function is applied, where the exact crossover point is also randomly picked. Consequently, the generated offspring is a combination of parts from both parent molecules, where the number of atoms and atom types are conserved. If the constraint of a minimum interatomic distance of 0.6 Å between any two atoms is not fulfilled in the generated structure, a different crossover point is used.

Lastly, the mutation function is applied, mutating each member of the current population with a probability of 0.02 for all three kinds of mutation. The mutation operations correspond to either switching the positions of two random atoms, randomly permuting the coordinates of a randomly chosen atom, or rotating a randomly chosen atom around all three molecular axes from the (30°, 60°) interval with the molecular center of mass being the center of rotation.

A combined energy and age-based filtering approach was implemented, which eliminates structures from the current population based on their similarity in electronic and nuclear repulsion energy, with the duplicate threshold values set to 10^−6^ a.u. and 10^−4^ a.u., respectively, as well as their age within the algorithm, set to a maximum of 5 cycles. This dual filtering technique further improves the population diversity and prevents stagnation on specific molecular configurations. For the final analysis, we consider all local minimum structures found during the GA run.

### Quantum chemical calculations

Among Ag^+^(H_2_O)_*n*_, *n* = 3–6, structures generated through GA, only structures with intact water molecules (“intact structures”) were considered further. All intact structures with energies below a threshold of 30 kJ mol^−1^ relative to their respective lowest-energy structure at the BLYP/def2SVP level were then re-optimized at the B3LYP/aug-cc-pVTZ-PP level of theory, followed by a frequency calculation. Additionally, single-point calculations of the electronic energy were performed at the CCSD(T)-F12A/aug-cc-pVTZ-PP^48^ level of theory using MOLPRO.^49–51^ The obtained relative energies are consistent with energies obtained from extrapolating CCSD(T)/aug-cc-pV*X*Z-PP (*X* = D, T) energies to the complete basis set (CBS) limit using a power function scheme, *E* = *E*_∞_ + *BX*^−3^, with *B* being a fit parameter and *X* = 2, 3 for DZ and TZ respectively.^52^ The results were then corrected for the zero-point energy obtained from the B3LYP calculations. Table S4† includes benchmarking against experiment and previous calculations, showing that CCSD(T) and CCSD(T)-F12A results are consistent irrespective of the method used for optimization, with bond energies for *n* = 1,2 lying somewhat below the experimental value.

### Master equation modeling

Temperature-dependent Blackbody Infrared Radiative Dissociation (BIRD) rate coefficients were calculated using master equation modeling (MEM) and subsequently compared with previously obtained experimental data^40^ through an Arrhenius plot. We applied both a single-well approach, where all molecules are assumed to be in the tentative global minima of their respective potential energy surface, as well as a multiple-well approach where several low-lying local minima are allowed to be populated as described in ref. 43.

In short, the molecules are modeled using the rigid-rotor harmonic oscillator approximation, where all vibrational modes are assumed to be active. For the inclusion of external rotations, the separable rotor approximation is used, allowing for an independent treatment of the rotors, where the 1D rotor (*K*-rotor), is assumed to be active and no limitations are imposed on the value of *K*, and the 2D rotor (*J*-rotor) is assumed to be inactive so that angular momentum is conserved during intramolecular vibrational redistribution (IVR).^53^ The MEM procedure then starts by assuming a Boltzmann distribution at ambient temperature for the energy distribution. Within each energy bin with a width of Δ*E* = 50 cm^−1^, the population is distributed over all isomers according to their density of states. Population can then be transferred from one bin to another *via* IR emission/absorption. As a third process, dissociation can occur if the internal energy is greater than the activation energy *E*_A_. This microscopic unimolecular dissociation rate coefficient can be expressed using Rice–Ramsperger–Kassel–Marcus (RRKM) theory,1
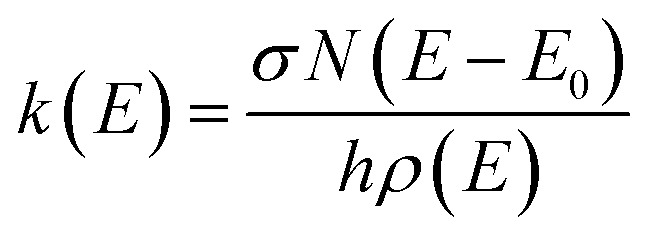
where *N*(*E* − *E*_0_) corresponds to the ro-vibrational sum of states of the transition state (TS), *ρ*(*E*) is the ro-vibrational density of states of the reactant, *h* is Planck's constant, and *σ* denotes the reaction pathway degeneracy. There are several approaches within the RRKM framework on how to model the transitional modes, meaning how to treat vibrational modes of the reactant that turn into rotations of the product during dissociation,^54^ which is crucial for the calculation of *N*. Here, in the single well limit, two different approaches were applied: (a) Rigid activated complex (RAC) RRKM,^55^ also known as tight transition state (TS), treating transitional modes as vibrations for a given TS, implying the need for an explicit definition of the TS. In our practical implementation, we take the TS structure as the structure of the reactant, removing the vibrational mode which corresponds to the reaction coordinate, and lifting the structure's energy by the dissociation energy. (b) Phase space limit (PSL), also known as loose TS. Here, transitional modes are taken as external rotations of the products without considering angular momentum conservation. The TS is then modeled to be the reaction outcome, *i.e.* two dissociated molecules. We closely follow the implementation of Rodgers, Ervin and Armentrout.^56^ Note that in both cases, no TS optimization is necessary, because the loose TS consists of the optimized structures of the fragments.

For multiple-well MEM, we use the *All Wells and Transition States Are Relevant* (AWATAR, loose TS) version^57^ of RRKM theory, where all energetically accessible isomers are considered according to2
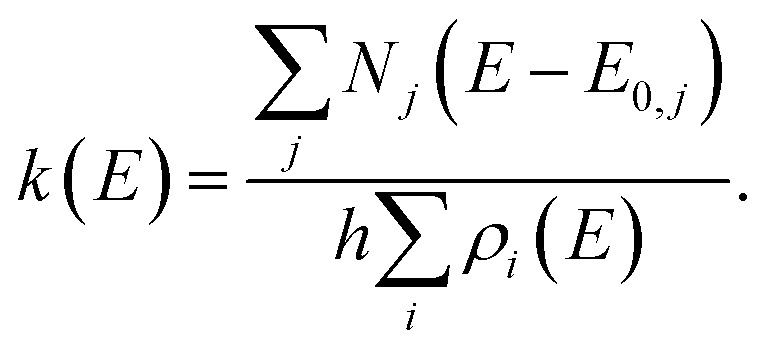
Here the density of states *ρ*_*i*_(*E*) at energy *E* is summed over all reactant local minima *i*, and the sum of states *N*_*j*_(*E* − *E*_0,*j*_) with barrier *E*_0,*j*_ is summed over all transition states *j*, *i.e.* all isomers of Ag^+^(H_2_O)_*n*–1_ in combination with an H_2_O molecule. Further, the implementation of the MEM protocol according to ref. 43 results in additional parameters that need to be specified. We introduce an empirical IR intensity scaling factor *s* that controls IR intensities and mostly introduces a constant offset of the modeled dissociation rate coefficients from the experimental ones, see Fig. S1 in the ESI.† Unless stated otherwise, this parameter is set to *s* = 1, which refers to no scaling. Additionally, the temperature of the ICR cell window and its covered solid angle are specified as 288 K (room temperature) and 0.2%, respectively. The induced effect of changing the solid angle on the final rate coefficients is small, see Fig. S2.†

For the Arrhenius plot, the standard Arrhenius eqn (3) was fitted to the calculated rate coefficients. Here, *k* represents the rate coefficient, *A* is the pre-exponential frequency factor, *T* is the absolute temperature, *E*_A_ is the activation energy, and *R* is the ideal gas constant.3
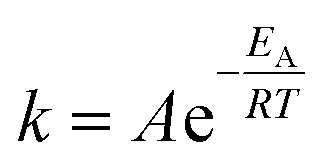


Additionally, a modified Arrhenius eqn (4) was utilized. In this equation, a temperature-dependent pre-exponential factor is introduced through the temperature exponent *χ*. Unlike the classical Arrhenius behavior, this allows the introduction of curvature into the plot.4
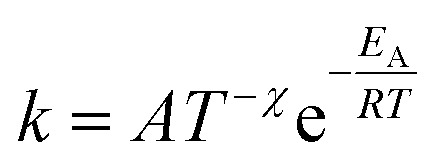


### Molecular dynamics

The dynamics of solvated silver clusters are analyzed using molecular dynamics (MD) simulations performed within the ABIN program,^58,59^ with forces and energies calculated using the BLYP/def2SVP method. The lowest-energy structure of Ag^+^(H_2_O)_4_ was simulated at five internal energies, 10, 20, 30, 40, and 50 kJ mol^−1^, using the microcanonical ensemble, with a time step of 20 a.u. (∼0.48 fs).

The obtained trajectories were analyzed by first eliminating any resulting rotational motion of the whole structure. This was achieved by aligning the trajectory to the initial frame using MDAnalysis^60^ while only considering the non-dissociating part of the molecule. The occupied volume of the dissociating water molecule was then estimated using a convex hull algorithm, as implemented in SciPy^61^ where the volume is defined as the space in which the oxygen atom with its van der Waals radius of 1.52 Å is moving over the simulated period.

To determine whether the simulation has converged, the trajectory was split into two parts. For each part, the volume in which the potentially dissociating water molecule can move freely was calculated. If the two calculated volumes agree within 2%, the simulation was considered to be converged, with simulation times of about 30 ps (see Table S1†).

## Results and discussion

### Geometry optimization

To locate the most important isomers of Ag^+^(H_2_O)_*n*_, *n* = 3–6, we used genetic algorithm optimization. The usage of the BLYP/def2SVP level of theory to efficiently evaluate the fitness of individual structures in the GA runs seems to be appropriate as mostly only a relative shift in energy and small changes to the structures could be observed when compared to the B3LYP/aug-cc-pVTZ-PP results (see Table S2†). The relative energetic ordering of the isomers did not change for *n* = 3 and 4, allowing to introduce a cutoff at 30 kJ mol^−1^ relative to the lowest energy structure for the cluster sizes at the BLYP level. This cutoff was also applied for *n* = 5 and 6 where the energetic ordering did not always stay the same, however, the relative energy shifts were small (Table S2†). As higher-lying isomers are not expected to be populated significantly at the experimental temperatures of 280 to 320 K, only isomers below the cutoff are considered for the RRKM calculations. From the pool of all structures, we considered only structures with intact water molecules as all other structures, *e.g.* an H_2_ molecule coordinated to AgO^+^(H_2_O)_*n*−1_, lie considerably higher in energy.

**Fig. 1:**
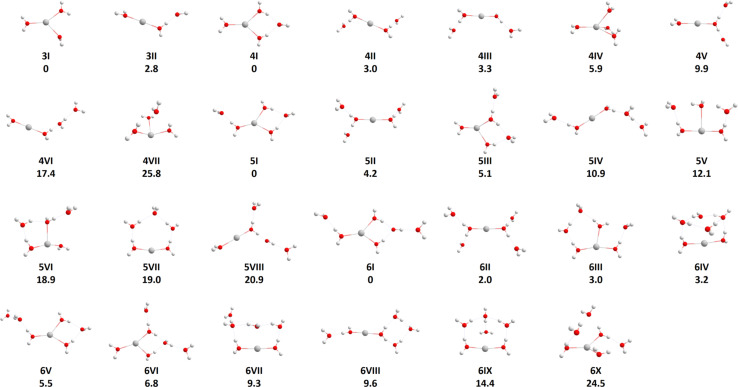
Optimized structures of Ag^+^(H_2_O)_*n*_, *n* = 3–6, at the B3LYP/aug-cc-pVTZ-PP level of theory. The electronic energy in kJ mol^−1^ relative to the lowest energy structure of each size has been evaluated using a single-point calculation at the CCSD(T)-F12A/aug-cc-pVTZ//B3LYP/aug-cc-pVTZ-PP level and is zero-point corrected.

The structures of Ag^+^(H_2_O)_*n*_, *n* = 3–6, which we used for the modeling, are included in Fig. 1. All isomers found are summarized in the ESI, Fig. S3.† For *n* = 3, the most stable isomer corresponds to a three-fold coordinated Ag^+^ (3I). An isomer with doubly-coordinated Ag^+^ and one water molecule in the second solvation layer lies about 3 kJ mol^−1^ higher in energy (3II), isomers with singly coordinated Ag^+^ lie considerably higher (see the ESI†). For *n* = 4, three-fold coordination is still preferred, with the fourth water molecule bound by two hydrogen bonds (4I). However, structures with the coordination number of two (4II, 4III) and four (4IV) lie close in energy, with structure (4IV) being the only one that had to be added manually as it could not be found by the GA. A similar pattern is also observed for *n* = 5 and 6, with the coordination number of three in the lowest lying isomers 5I and 6I again being slightly favored over other possibilities. For *n* = 5 and 6, all found clusters have coordination number two or three, we did not succeed in optimizing clusters with four-fold coordination as the coordination number decreased upon optimization. In the lower lying isomers for *n* = 4, 5 and 6, all water molecules are in the first or second hydration shell, but there are some higher lying isomers with water molecules in the third hydration shell, as for example in isomers (4VI) and (5IV). As expected, water evaporation energies decrease when moving from *n* = 4 (55.0 kJ mol^−1^) through *n* = 5 (47.0 kJ mol^−1^) to *n* = 6 (41.7 kJ mol^−1^) when the lowest-lying isomers are considered for each cluster size. The low-lying isomers for *n* = 3, 4 found by genetic algorithm runs correspond to the ones reported earlier, as already partially discussed in the Introduction. In agreement with Feller *et al.*,^21^ we could identify the (3 + 0) and (3 + 1) coordinated structures for *n* = 3 and *n* = 4, respectively, as the global minima. Further, as reported by Ohashi and co-workers^28^ hydrogen bonding can already be observed for *n* = 3 with the linear (2 + 1) coordination only slightly higher in energy than the (3 + 0) structure, and for *n* = 4 with (3 + 1) and (2 + 2) coordinated structures.

### Master equation modeling

The B3LYP optimized isomers were used for master equation modeling. For the single-well approach, the reaction channel of the most stable isomer *NI* (*N* = 4–6) dissociating to the most stable isomer with one water molecule less (*N* = 3–5) was considered. In the multiple-well approach, all energetically accessible reaction channels are included implicitly within the AWATAR approach through summation over all isomers as well as all transition states as shown in eqn (2). Thus, it is assumed that the barrier for interconversion between different isomers is small compared to the dissociation energy, which enables us to use the summed density of states for calculating the relative population. This assumption was recently shown to be valid for NaCl clusters.^57^

**Fig. 2:**
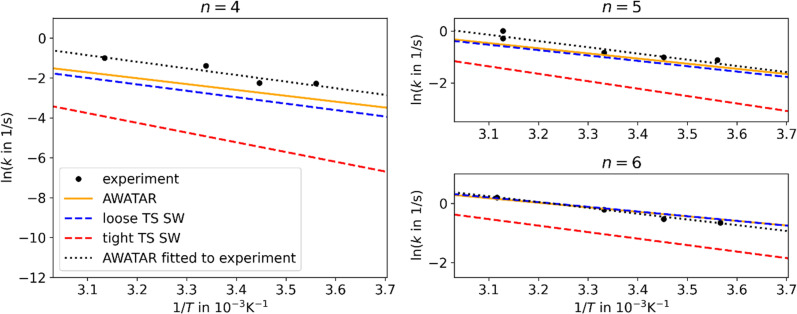
Arrhenius plots for Ag^+^(H_2_O)_*n*_, *n* = 4–6, comparing experimental results^40^ (black points) to linear fits of the rate coefficients as obtained from MEM within the AWATAR approach (orange) and single-well approach (blue and red dashed lines). The dotted black lines were obtained by fitting the activation energy and IR intensity within the AWATAR approach to the experimental data. For clarity, only linear fits of rate coefficients calculated in the range of 280 to 320 K are included in the plot.

In Fig. 2, we compare the experimental data to the different theoretical treatments of the transitional modes, namely the RAC-RRKM (tight TS) and PSL-RRKM (loose TS) for the single well approach, and the PSL-AWATAR (loose TS) for the multiple-well approach (see Fig. S6† for results for *n* = 3). Predicted dissociation energies are collected in Table 1. The experiment on Ag^+^(H_2_O)_*n*_, *n* = 4–6, clusters was performed in a cooled cell under the influence of BIRD, varying the temperature in the range of 160–320 K.^40^ Here, only temperatures down to 280 K are considered, as there is some evidence that the lower-temperature dissociation rate coefficients of this experiment series might be influenced by collisions due to the long lifetimes of the clusters at lower temperatures. It should be noted that the Ag^+^(H_2_O)_*n*_ BIRD experiments were among the first performed with the then newly constructed cooled ICR cell.^40^ Based on the scattering of the measured rate coefficients, we estimate the relative error of the slopes of the Arrhenius fits to be ∼25%.

**Table 1:** MEM activation Energy *E*_A,MEM_ as found by fitting the MEM rate coefficients to the Arrhenius plot of the experimental data for *n* = 4–6, see Fig. 2, and calculated dissociation energy *E*_A_ at CCSD(T)-F12A/aug-cc-pVTZ-PP//B3LYP/aug-cc-pVTZ-PP level with zero-point corrections. Calculated reaction enthalpies Δ*H* (*T* = 298 K, *p* = 1 atm) are compared to the experimental data from literature. *s*_fit_ is a fit parameter that controls the IR absorption intensities and SW/MW indicates the single-well and multiple-well MEM approach, respectively

*N*	4	5	6
*E*_A,MEM_ (SW, tight TS) [kJ mol^−1^]	43	42	39
*E*_A,MEM_ (SW, loose TS) [kJ mol^−1^]	56	52	50
*E*_A,MEM_ (MW, AWATAR) [kJ mol^−1^]	59	53	51
*E*_A,CCSD(T)-F12A_ [kJ mol^−1^]	55.0	47.0	41.7
Δ*H*_A,CCSD(T)-F12A, 298K_ [kJ mol^−1^]	58.9	48.9	43.6
Δ*H*_A_ (ref. 19) [kJ mol^−1^]	51 ± 12	—	—
Δ*H*_A_ (ref. 18) [kJ mol^−1^]	62 ± 1	57 ± 1	56 ± 3
*s*_fit_ (SW, tight TS)	3.9	2.4	1.9
*s*_fit_ (SW, loose TS)	3.8	2.4	1.9
*s*_fit_ (MW, AWATAR)	3.8	2.4	2.0

**Fig. 3:**
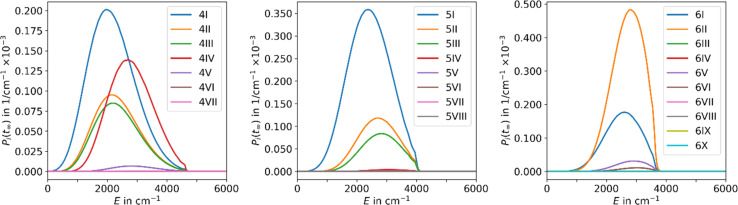
Population of the energy levels of all isomers included in MEM at the stationary point *P*_*i*_(*t*_inf_) for Ag^+^(H_2_O)_*n*_, *n* = 4–6, at 280 K in the AWATAR approach.

The results obtained using the tight TS approach predict considerably lower reaction rate coefficients compared to both loose TS approach and experimental data, by up to three orders of magnitude, as well as a different slope (Fig. 2). On the other hand, the loose TS approach provides results that are much closer to the experimental measurements already for a single well. Using multiple wells within loose TS in the AWATAR ansatz further increases the rate coefficients towards the experimental ones for *n* = 4, but does not affect the results considerably for *n* = 5, 6. The reason for this behavior can be understood by inspection of the relative isomer populations at *T* = 280 K, see Fig. 3. For *n* = 4, isomer 4IV is strongly populated close to the dissociation energy threshold and contributes thus considerably to the overall rate coefficient. Omitting this single isomer leads to underestimation of the overall rate coefficient (see also Fig. S4†). For *n* = 5, 6, other than most stable isomers become also considerably populated; however, they do not shift the total population that much to higher energies, and thus the dissociation rate coefficients are not markedly influenced.

For all investigated cluster sizes, loose TS approaches are in a quantitative agreement with the experimental BIRD rate coefficients, with a slight deviation of the slope of the Arrhenius plots (Table S3†). By manually shifting the activation energies and the IR intensity scaling factor *s* of the isomers to match the experimental slope, we approach the experimental activation energy. In Fig. 2, the dotted black lines indicate this fit for the AWATAR approach, all fits of activation energies and IR absorption intensities are shown in Table 1. For the AWATAR approach, the fitted values of the activation energy lie within 4–9 kJ mol^−1^ above the value determined by the CCSD(T)-F12A approach, within the expected accuracy.

In Table 1, we also compare the modeled values to the other experimental data. The experiments by Armentrout *et al.*^19^ for *n* = 4 are consistent with both CCSD(T)-F12A values and MEM fits within the reported experimental uncertainties. The experiments by Holland and Castleman predict considerably higher dissociation enthalpy values,^18^ lying up to 13 kJ mol^−1^ above the calculated ones. At the same time, while our CCSD(T)-F12A calculations and the fitted dissociation energies predict a shift of ∼13 kJ mol^−1^ and ∼8 kJ mol^−1^ between *n* = 4 and *n* = 6, respectively, a shift of ∼6 kJ mol^−1^ in enthalpy was measured by Holland and Castleman.^18^ However, their values are still consistent with the energies deduced from master equation modeling (note that at 298 K, the dissociation enthalpy is by ∼2–4 kJ mol^−1^ higher than the dissociation energy here, see Table 1).

**Fig. 4:**
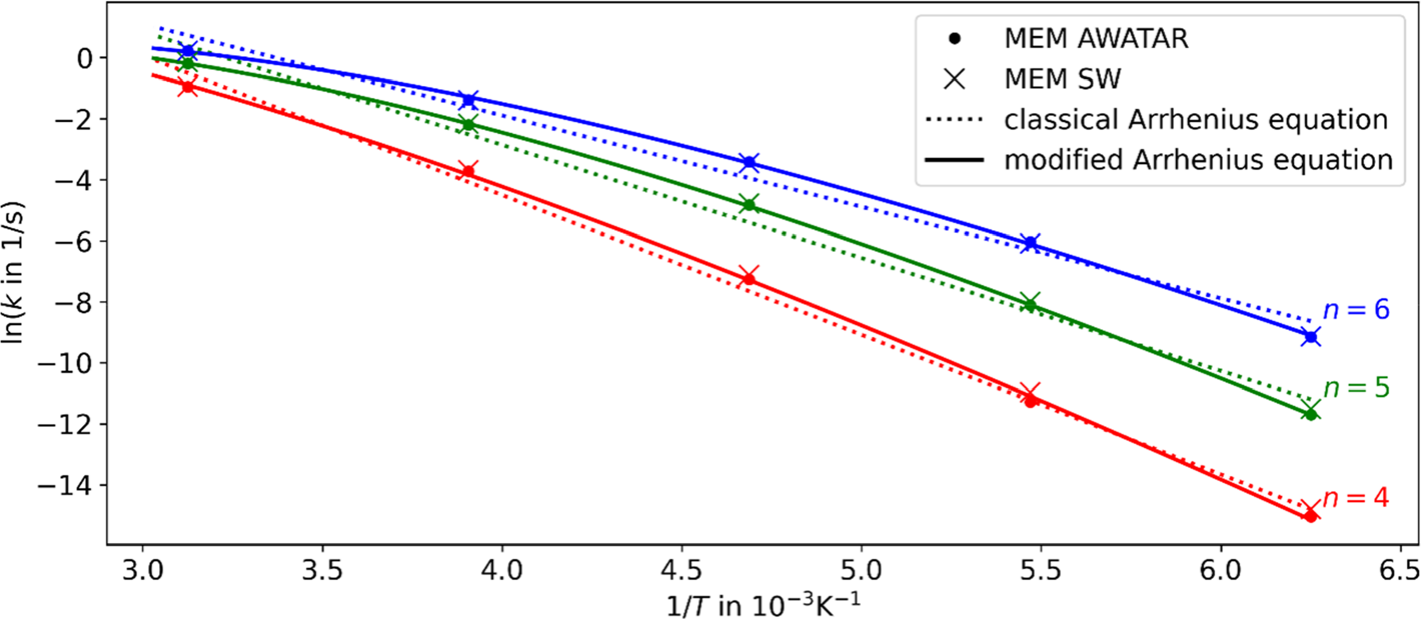
Arrhenius plots for *n* = 4, 5, and 6 using the AWATAR and single-well (SW) approach within loose transition state approximation. The MEM rate coefficients as fitted to the experiment have been used. The dotted lines represent the linear fits using a classical Arrhenius equation and the solid lines indicate the fits using the modified Arrhenius equation. For the fits, the AWATAR data has been used; the SW data is only shown for comparison. A curvature with *χ* = 12.5, 18.3, 17.1 for *n* = 4, 5, 6, respectively, can be observed.

**Fig. 5:**
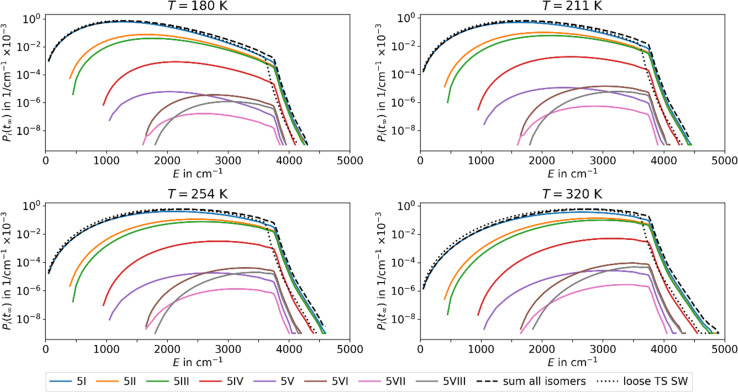
Population of the energy levels of all isomers included in MEM at the stationary point *P*_*i*_(*t*_inf_) at different temperatures *T* for Ag^+^(H_2_O)_5_ in the AWATAR approach. Activation energies and scaling factors are given in Table 1. The dashed lines indicate the sum of the population for all isomers in the AWATAR approach, and the dotted lines the population for the loose TS SW approach.

Using the fit parameters Δ*E*_A,fit_, *s*_fit_ and *χ* we model MEM rate coefficients over a wider temperature range of 180–320 K and fit them to the modified Arrhenius equation eqn (4). A distinct curvature can be observed, see Fig. 4. This illustrates the limitations of the classical Arrhenius equation, which assumes a linear behavior. The difference is quantified by the non-zero temperature exponents of *χ* = 12.5, 18.3, and 17.1 for *n* = 4, 5, and 6, respectively. In Fig. 4, full lines represent fits using the modified Arrhenius equation, while dotted lines denote the classical Arrhenius equation. The curvature can be explained by the extent of truncation of the Boltzmann distribution as a function of temperature, see Fig. 5. For low temperatures, only a small fraction of the Boltzmann distribution is truncated, as most of the molecules have energies below the activation barrier. Therefore, modelling the population as a Boltzmann distribution is a reasonable approximation, the Arrhenius equation holds, and the relation between the logarithm of the rate coefficient and the inverse of the temperature is approximately linear. For higher temperatures, on the other hand, the truncation of the Boltzmann distribution is getting more pronounced. Therefore, the Arrhenius approximation breaks down and the actual population is much lower than the Boltzmann distribution would predict. Therefore, for higher temperatures, the rate coefficients are lower than expected from a linear fit (Fig. 4).

The inclusion of several isomers in the multi-well AWATAR MEM further increases the non-linearity slightly. This is due to the fact that when increasing the temperature, higher-lying isomers can be populationed more easily, and the availability of higher-lying isomers is not linear. However, this is only a minor effect compared to the curvature induced by the MEM approach *per se*.

The results of the MD simulations on Ag^+^(H_2_O)_4_, isomer 4I, show no isomerization in the investigated energy range of 10 kJ mol^−1^ to 50 kJ mol^−1^, most probably due to the limited simulation time. However, the results illustrate nicely that, with an increase in internal energy, the occupied volume of the dissociating water molecule increases in a gradual fashion, see Fig. S5 and Table S1 in the ESI.† Such a behavior is expected from a system of loosely bound water molecules.

## Conclusion

In this study, we examined the dissociation rate coefficients of water molecules from Ag^+^(H_2_O)_*n*_, *n* = 4–6, clusters within blackbody infrared radiative dissociation. We employed both single-well and multiple-well approach, in combination with a genetic algorithm, ensuring an efficient and systematic exploration of the potential energy surface. For the calculation of unimolecular rate coefficients, we evaluated two distinct methods based on RRKM theory: the rigid activated complex (“tight TS”) and the phase space limit approach (“loose TS”). The two approaches can be viewed as two simplified treatments of transition state position. Our findings suggest that the phase space limit provides a more accurate depiction of transitional modes for solvated silver cations.

Through fitting the experimental data, we obtained the activation energy of water dissociation reactions for *n* = 4–6. This revealed an error of up to ∼9 kJ mol^−1^ in activation barrier between the MEM approach based on FT-ICR measurements, and quantum chemical calculations using the CCSD(T)-F12A method, well within the expected accuracy and in agreement with previous experiments. By applying a modified Arrhenius equation with a temperature-dependent prefactor, our results predict a deviation from the classical Arrhenius behavior, induced by a pronounced curvature over the examined broad temperature range. It became apparent that isomers positioned at higher energy levels might have a significant impact on the overall MEM rate coefficients, emphasizing the importance of the multiple-well MEM treatment. Without considering higher-lying isomers in the kinetic modeling, one might easily overlook energetically higher-lying structures that still contribute significantly to the observed kinetics. The AWATAR approach treats all isomers on the same footing, without the need to define *a priori* neither the most important isomers nor specific dissociation pathways, qualifying thus as a method of choice for treating dissociation dynamics in loosely bound molecular clusters.

## Data availability

The data supporting this article have been included as part of the ESI.†

## Conflicts of interest

There are no conflicts to declare.

## Supplementary Material

RA-014-D4RA03518F-s001
