# Supplementary material for: Master equation modeling of water dissociation in small ionic water clusters: Ag+(H2O)n, n = 4–6
Source: RSC Adv. 2024 Jul 12;14(31):22185–94. doi: 10.1039/d4ra03518f (PMC11244579; doi:10.1039/d4ra03518f)
Supplement: RA-014-D4RA03518F-s001 [file RA-014-D4RA03518F-s001.pdf]

Electronic Supplementary Material (ESI) for RSC Advances.
This journal is © The Royal Society of Chemistry 2024

Supporting Information

Master Equation Modeling of Water Dissociation in Small Ions: $Ag^+(H_2O)_n$, *n* = 4–6

Michael Hütter, Gabriel Schöpfer, Magdalena Salzburger, Martin K. Beyer, and Milan Ončák**

Institut für Ionenphysik und Angewandte Physik, Universität Innsbruck, Technikerstraße 25, 6020 Innsbruck, Austria

E-Mail: Martin.Beyer@uibk.ac.at, Milan.Oncak@uibk.ac.at

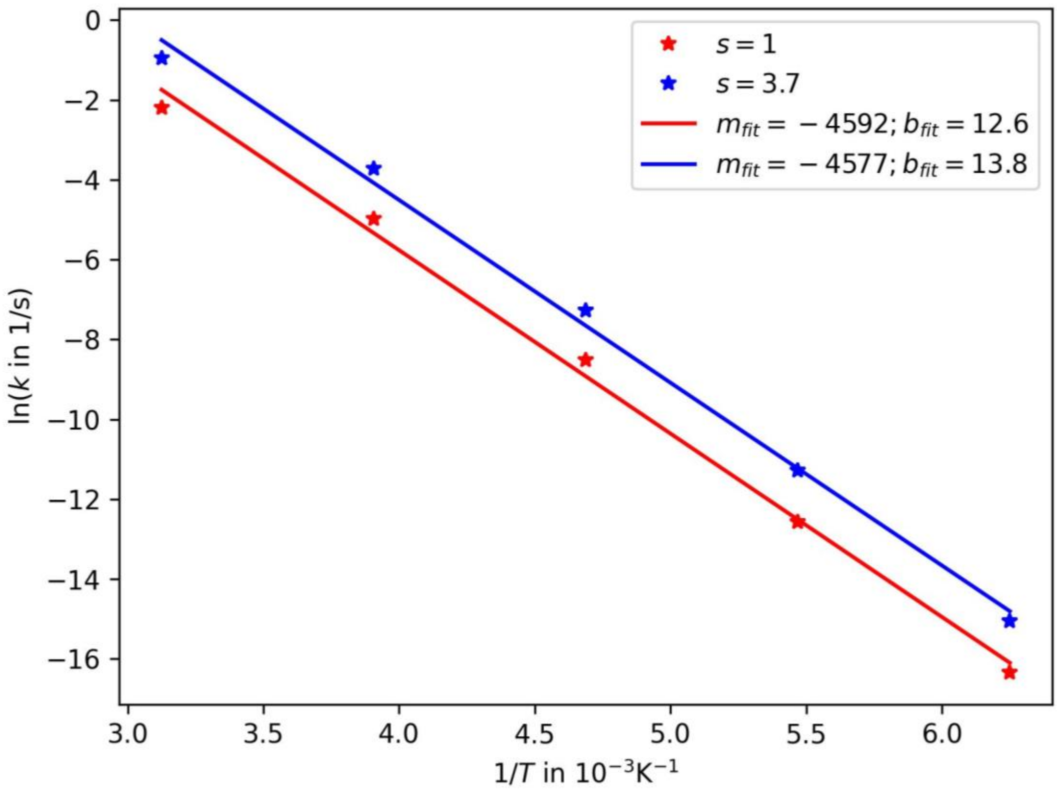

Figure S1: Effect of the empirical scaling factor s, controlling the IR intensities, on the slope *m* and constant offset *b* as introduced in the Arrhenius plot. Changing s mostly introduces a constant offset of the modeled MEM rate coefficients and only has a small effect on the resulting slope as fitted to the Arrhenius equation, see m_{fit} and b_{fit}. Illustrated for *n* = 4 in the AWATAR approach for a temperature range of 160 K to 320 K.

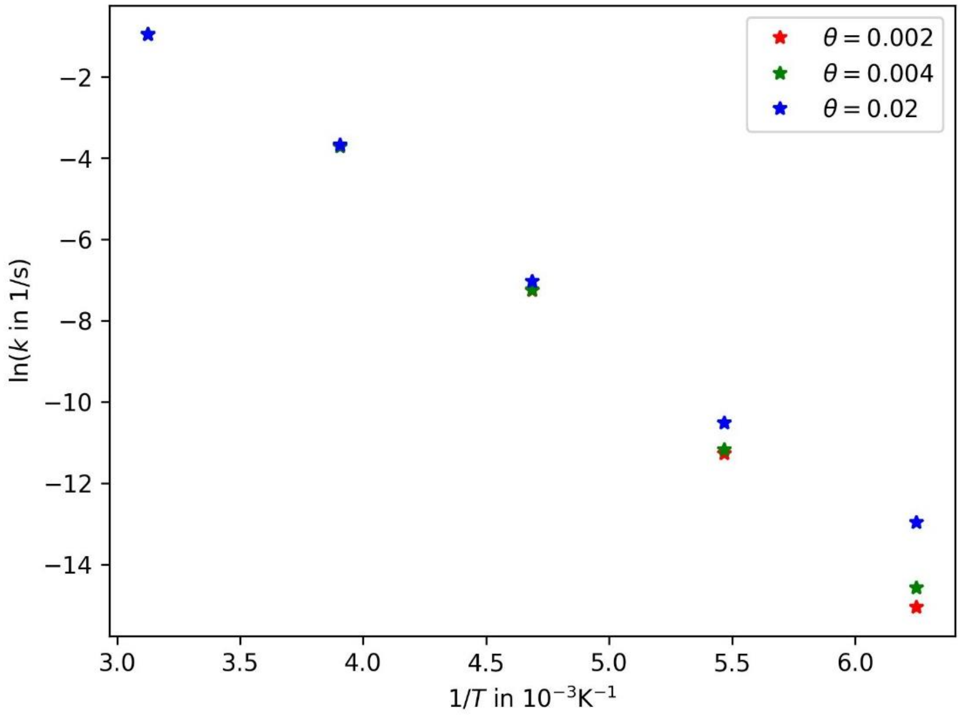

Figure S2: Rate coefficients for three different solid angle values (to model the influence of the ICR window, see Methods) illustrated for n = 4 in the AWATAR approach. Differences become only important below 210 K, above this temperature, the difference in the logarithmic rate coefficients stays below 3%.

Table S1: Results of the MD simulations for isomer **4l**. E is the internal energy, ΔE the total energy shift over the time period t, corresponding to the total simulation time with a timestep of 20 a.u. (~0.48 fs). V is the calculated volume in which the oxygen atom of the weakest bound water molecule was free to move over the simulation and ε refers to the relative error in the volume convergence.

E in kJ/mol	ΔE in meV/(atom×ps)	t in ps	V in Å^3	ε in %
10.4	7.1	30	24.8	1.3
20.3	5.4	29	34.8	1.9
29.7	3.8	29	41.5	0.5
39.8	5.2	28	43.9	0.9
49.6	2.8	28	47.2	1.0

Table S2: Comparison of the electronic energy of the isomers in Figure 1 at different levels of theory. For BLYP and B3LYP, the clusters were optimized at the respective levels. For CCSD(T)-F12A, single-point calculations in the B3LYP optimized structures were used, along with B3LYP zero-point energy correction. Unless noted otherwise, the aug-cc-pVTZ-PP basis set was used. Energy in kJ/mol. Structure 4IV was the only one that had to be added manually, as no such structure could be found by the genetic algorithm at the BLYP/def2SVP level of theory.

Isomer	BLYP/def2SVP	B3LYP	CCSD(T)-F12A	CCSD(T)/CBS
3I	10.8	7.6	0.0	0.0
3II	0.0	0.0	2.8	1.3
4I	12.5	12.1	0.0	0.0
4II	0.0	0.0	3.0	1.2
4III	1.2	0.4	3.3	1.5
4IV	–	23.8	5.9	6.1
4V	10.9	8.3	9.9	8.2
4VI	17.0	14.4	17.4	15.6
4VII	44.7	45.5	25.8	25.7
5I	8.5	9.8	0.0	0.0
5II	0.0	0.0	4.2	6.8
5III	14.9	15.2	5.1	4.9
5IV	5.1	5.1	10.9	13.8
5V	7.6	20.3	12.1	10.5
5VI	13.7	26.1	18.9	18.3
5VII	7.4	18.7	19.0	19.4
5VIII	17.6	17.0	20.9	22.8
6I	9.5	10.7	0.0	0.0
6II	2.9	0.0	2.0	0.4
6III	9.2	16.6	3.0	2.6
6IV	1.4	20.9	3.2	1.2
6V	18.0	15.9	5.5	5.6
6VI	18.6	17.9	6.8	6.9
6VII	10.5	17.8	9.3	7.9
6VIII	0.0	9.7	9.6	7.8
6IX	9.6	23.8	14.4	12.4
6X	28.1	47.0	24.5	24.1

$Ag^{+}(H_2O)_3$

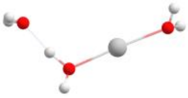

S3I
0

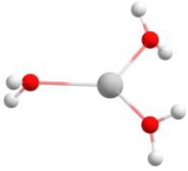

S3II
10.8

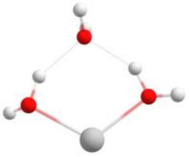

S3III
37.8

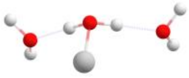

S3IV
70.9

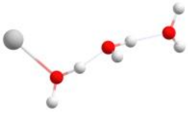

S3V
78.9

$Ag^{+}(H_2O)_4$

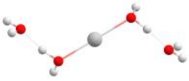

S4I
0

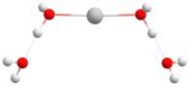

S4II
1.2

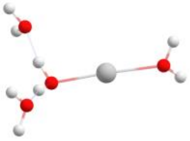

S4III
10.9

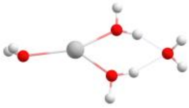

S4IV
12.5

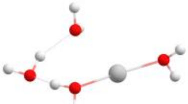

S4V
16.9

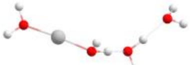

S4VI
17.0

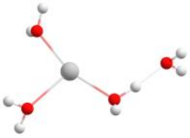

S4VII
18.7

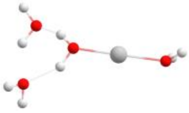

S4VIII
18.9

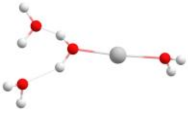

S4IX
19.1

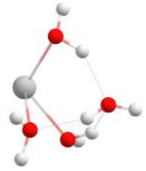

S4X
44.7

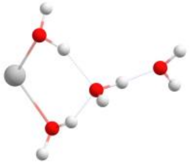

S4XI
47.3

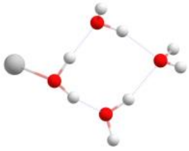

S4XII
80.5

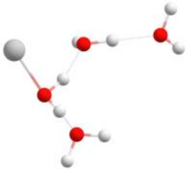

S4XIII
90.4

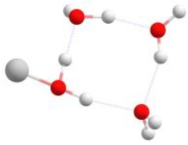

S4XIV
90.5

$Ag^+(H_2O)_5$

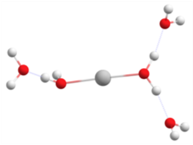

S5I
0

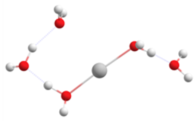

S5II
4.8

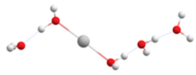

S5III
5.1

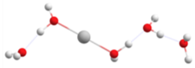

S5IV
5.3

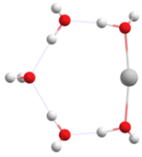

S5V
7.4

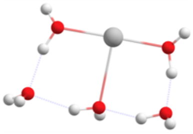

S5VI
7.6

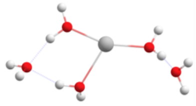

S5VII
8.5

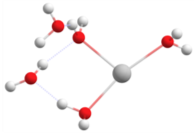

S5VIII
13.7

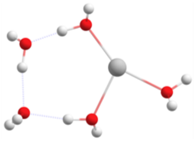

S5IX
14.3

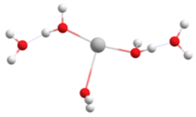

S5X
14.5

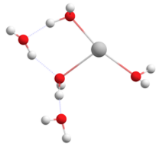

S5XI
14.9

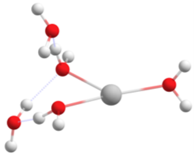

S5XII
16.3

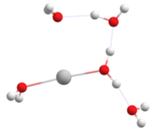

S5XIII
17.6

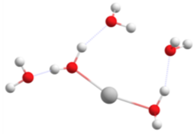

S5XIV
19.2

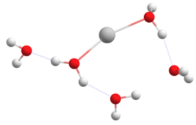

S5XV
19.4

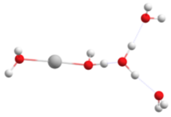

S5XVI
27.7

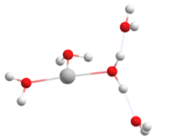

S5XVII
27.8

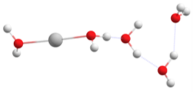

S5XVIII
28.8

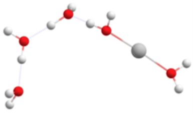

S5XIX
32.6

Ag^{+}(H$_2$O)$_6$

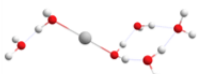
S6I
0.0

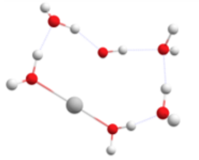
S6II
0.4

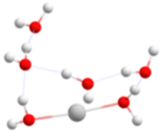
S6III
1.4

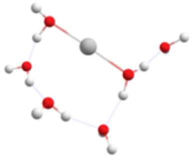
S6IV
2.6

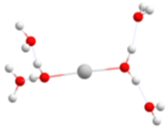
S6V
2.9

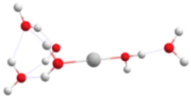
S6VI
4.8

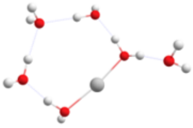
S6VII
6.6

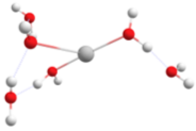
S6VIII
7.8

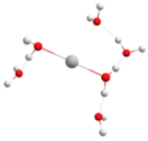
S6IX
9.1

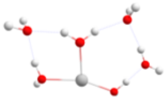
S6X
9.2

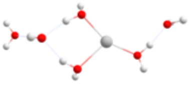
S6XI
9.5

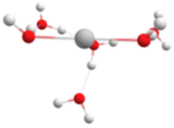
S6XII
9.6

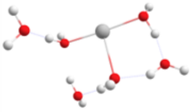
S6XIII
10.5

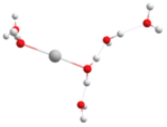
S6XIV
11.7

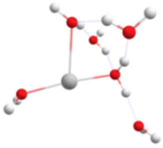
S6XV
11.8

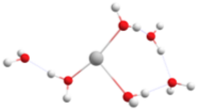
S6XVI
12.1

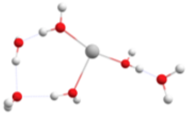
S6XVII
12.7

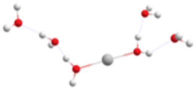
S6XVIII
13.9

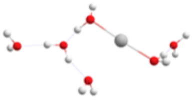
S6XIX
15.3

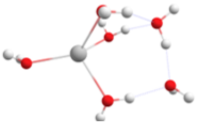
S6XX
17.5

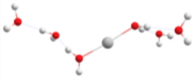
S6XXI
15.8

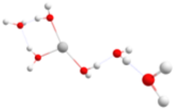
S6XXII
18.0

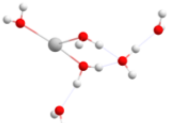
S6XXIII
18.6

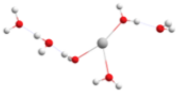
S6XXIV
25.9

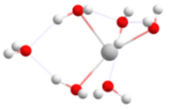
S6XXV
28.1

Figure S3: Intact structures as obtained from genetic algorithm runs at the BLYP/def2SVP level, along with relative zero point corrected electronic energy in kJ/mol.

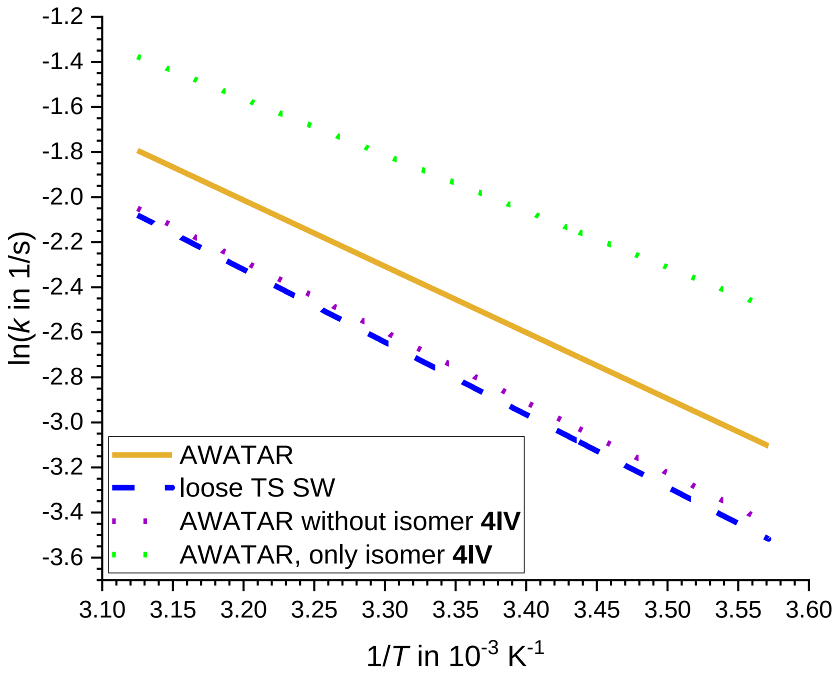

Figure S4: Influence of inclusion of isomer **4IV** on the dissociation rate coefficient: When isomer **4IV** is removed from the AWATAR approach (purple dotted line) , the results are very similar to the single well approach (blue dashed line). The green dotted line shows the results if all reactants but isomer **4IV** are neglected.

Table S3: Slopes *m* as found in the Arrhenius plot by the linear fits of the calculated rate coefficients for *n* = 4–6, see Figure 2.

N	4	5	6
m(SW, tight TS) [k]	-4900	-2900	-2200
m(SW, loose TS) [k]	-3200	-2100	-1600
m(AWATAR) [k]	-2900	-2000	-1500
m_{exp} [k]	-3300(800)	-2400(600)	-2000(500)

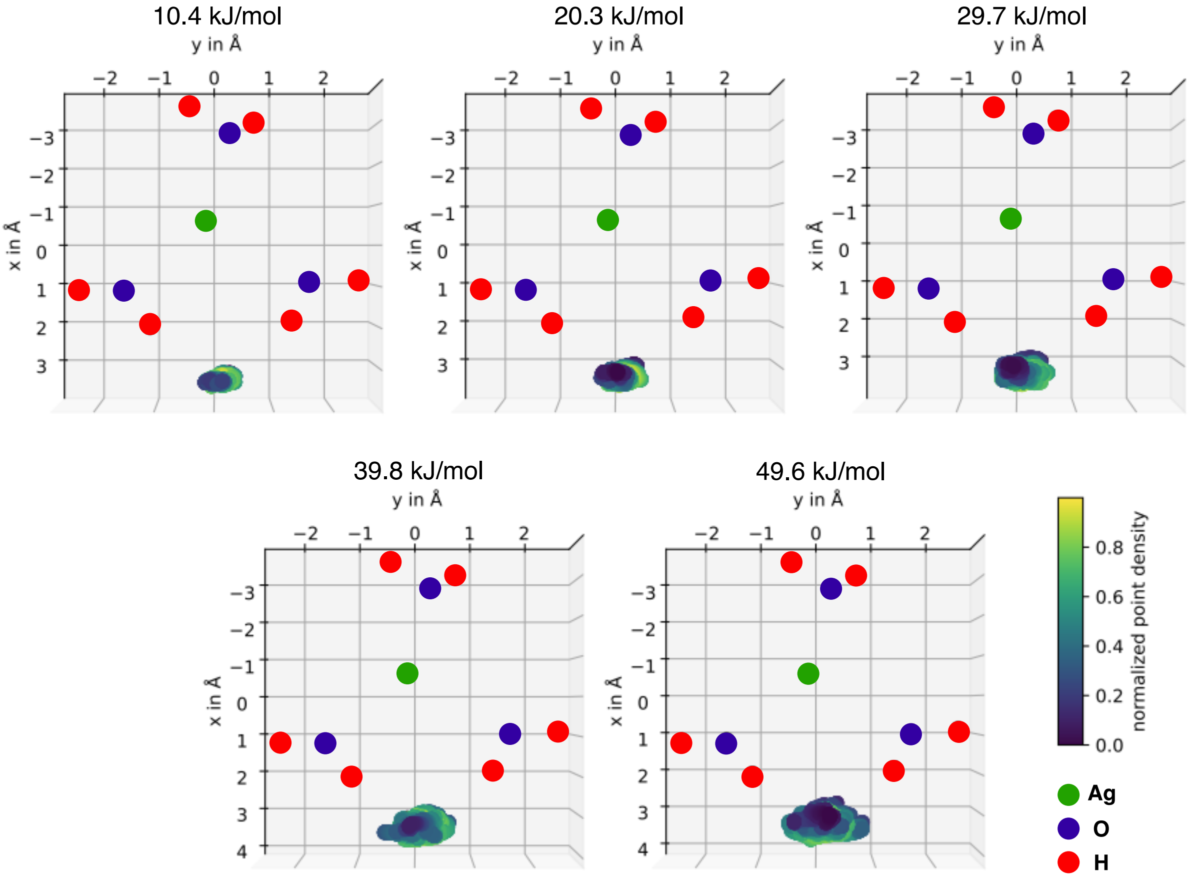

Figure S5: Resulting MD trajectories for $Ag^+(H_2O)_4$, isomer **4I**, for different internal energies. The normalized point density was obtained from a kernel density estimation with a Gaussian kernel, a bandwidth of 0.5, and is proportional to the number of data points that correspond to this specific configuration as obtained from the trajectories.

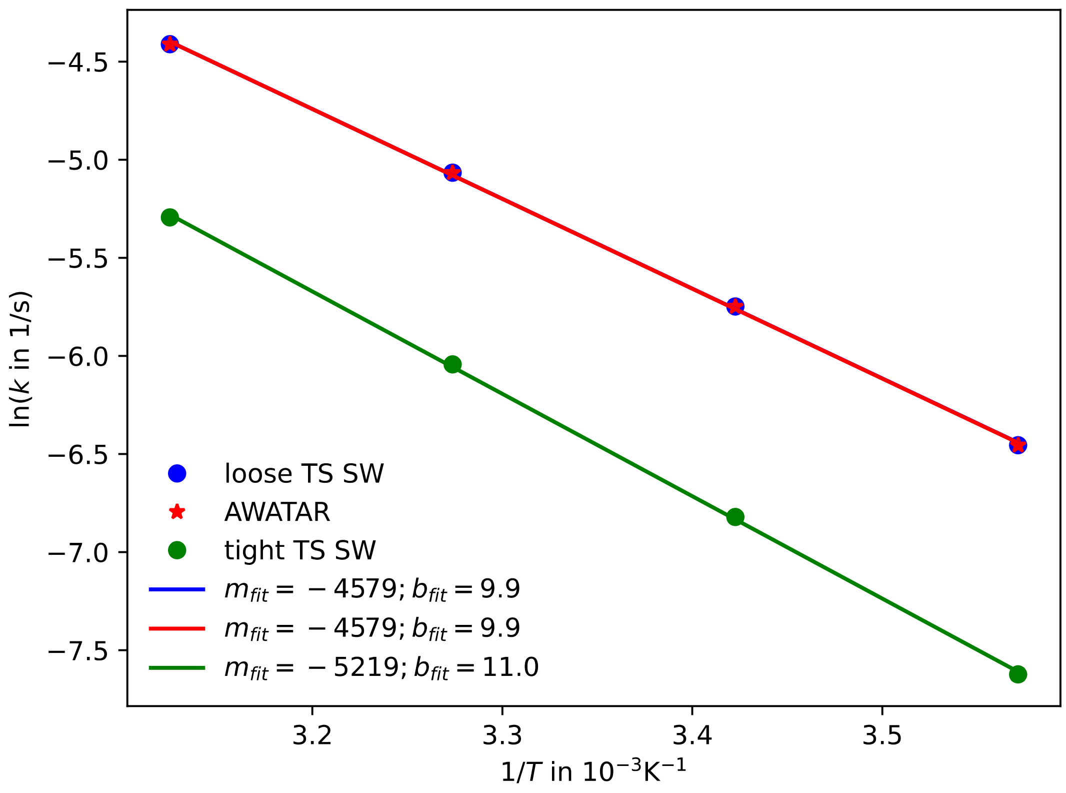

Figure S6: Arrhenius plot for $Ag^+(H_2O)_3$, with the MEM rate coefficients (dots and asterisks) and the resulting linear fits (continuous lines) for the AWATAR approach (green) and single-well approach (blue and red lines). Note that visually no difference in the MEM rate coefficients and resulting fits for the AWATAR approach and the loose TS SW approach can be seen.

Table S4: Bond dissociation enthalpy (in kJ/mol) for $Ag^+(H_2O)_n$ calculated using different levels of theory. "*XZ*" stands for aug-cc-pV*XZ*(-PP) basis set.

n	1	2	3
B3LYP/DZ	127	118	61
CCSD(T)/DZ//B3LYP/DZ	117	109	60
CCSD(T)-F12A/TZ//B3LYP/DZ	123	114	60
B3LYP/TZ	127	117	60
CCSD(T)/TZ//B3LYP/TZ	121	113	60
CCSD(T)-F12A/TZ//B3LYP/TZ	123	114	60
B3LYP/QZ	127	118	60
CCSD(T)/QZ//B3LYP/QZ	123	114	-
CCSD(T)-F12A/TZ//B3LYP/QZ	123	114	60
M06/TZ	123	114	59
CCSD(T)/TZ//M06/TZ	121	113	60
CCSD(T)-F12A/TZ//M06/TZ	123	115	60
TPSSh/TZ	126	119	62
CCSD(T)/TZ//TPSSh/TZ	120	112	60
CCSD(T)-F12A/TZ//TPSSh/TZ	123	114	59
CCSD(T)/DZ	111	-	-
CCSD(T)-F12A/TZ//CCSD(T)/DZ	123	-	-
CCSD/DZ	115	106	-
CCSD(T)/DZ//CCSD/DZ	117	110	-
CCSD(T)-F12A/TZ//CCSD/DZ	123	114	-
CCSD/TZ	118	108	-
CCSD(T)/TZ//CCSD/TZ	121	113	-
CCSD(T)-F12A/TZ//CCSD/TZ	123	115	-
CCSD/QZ	119	-	-
CCSD(T)/QZ//CCSD/QZ	123	-	-
CCSD(T)-F12A/TZ//CCSD/QZ	123	-	-
experiment [a]	134 ± 8	127 ± 8	63 ± 9
theory [b]	133	117	52

[a] – *Int. J. Mass Spectrom.* 228 (2003) 221–235.

[b] – *J. Chem. Phys.* 110 (1999) 1475–1491.

Cartesian coordinates of the isomers considered in the MEM calculations with their zero-point corrected electronic energies in a.u. (B3LYP/aug-cc-pVTZ)

```
2I: E = -299.661817
Ag 0.000000 0.000000 0.004168
O 0.000000 2.167732 -0.033569
H 0.404726 2.719271 0.646398
H -0.674623 2.696698 -0.475788
O -0.000000 -2.167732 -0.033569
H -0.404726 -2.719271 0.646398
H 0.674623 -2.696698 -0.475788
```

```
2II: E = -299.641450
Ag -0.929494 -0.112120 0.001010
O 0.961886 0.931474 -0.045274
H 1.839303 0.457427 0.006614
H 1.073049 1.834138 0.270854
O 3.193982 -0.429430 0.001776
H 3.735857 -0.609853 0.778083
H 3.791066 -0.428448 -0.755059
```

```
3I: E = -376.125701
O              -2.3944170000      0.0753700000     -0.0440660000
H              -2.9902610000     -0.6765440000      0.0472740000
H              -2.8935610000      0.8631980000      0.1982810000
Ag             -0.1631500000     -0.0794420000      0.0034400000
O               1.6649340000     -1.4448200000      0.0346290000
H               1.7050540000     -2.3960270000     -0.1132170000
H               2.5322530000     -1.0845110000     -0.1811380000
O               1.4796450000      1.6880330000     -0.0118870000
H               1.7518900000      2.1528850000      0.7879640000
H               1.5613690000      2.3261370000     -0.7302370000
```

```
3II: E = -376.128586
Ag              0.5351000000      0.1173580000     -0.0045700000
H               2.9467300000     -1.1719900000     -0.6807690000
O               2.5832950000     -0.5927390000     -0.0010490000
H               3.0893670000     -0.7383770000      0.8067320000
H              -4.0245910000     -1.0322610000     -0.7606330000
O              -3.5209590000     -0.7549710000      0.0123630000
H              -4.1232230000     -0.8016600000      0.7631450000
H              -1.6744140000      1.7344970000     -0.3260640000
O              -1.4512030000      0.8735760000      0.0410250000
H              -2.2526390000      0.2870330000     -0.0063480000
```

```
4I: E = -452.589669
O              -3.4360800000      0.0144160000     -0.0083690000
H              -4.0152760000     -0.0491880000     -0.7778200000
H              -4.0229620000      0.0847850000      0.7546320000
O               2.8692220000      0.0305980000     -0.0531570000
H               3.4042690000      0.7464410000      0.3065430000
H               3.4243540000     -0.7566090000     -0.0663930000
Ag              0.6189050000     -0.0106120000      0.0048370000
O              -1.0692490000      1.5913280000      0.0497670000
H              -1.0388580000      2.4890930000     -0.2949660000
H              -1.9813430000      1.2658060000     -0.0401360000
O              -1.0872670000     -1.5798950000     -0.0409900000
H              -1.9971060000     -1.2447220000      0.0376620000
H              -1.0746010000     -2.4884100000      0.2751410000
```

```
4II: E = -452.594263
Ag              0.0000920000     -0.2936470000      0.0003910000
H               2.7160800000      0.1518640000     -0.3933610000
H               2.2617720000     -0.9715400000     -1.4175280000
H              -4.3812510000      1.7011930000     -0.0392120000
H              -4.5504070000      0.5318830000     -1.0337040000
H              -2.2614650000     -0.9663900000      1.4209570000
H              -2.7159870000      0.1533000000      0.3929590000
H               4.5500040000      0.5358560000      1.0323190000
H               4.3801580000      1.7019210000      0.0341400000
O               1.9426870000     -0.2500640000     -0.8671240000
```

O	-3.9346800000	0.9328680000	-0.4111020000
O	-1.9424440000	-0.2469160000	0.8678860000
O	3.9340310000	0.9345260000	0.4084740000

4III: E = -452.594123

Ag	-0.0002220000	-0.4394590000	0.0000090000
O	3.7134960000	1.4250160000	-0.2998970000
H	3.9753290000	2.2096570000	0.1934890000
H	4.4350890000	1.2305770000	-0.9079370000
O	2.0661600000	-0.4827980000	0.5052160000
H	2.6975440000	0.2383320000	0.2504660000
H	2.3766790000	-0.8917640000	1.3185190000
O	-2.0667080000	-0.4834510000	-0.5048560000
H	-2.3769010000	-0.8920310000	-1.3184800000
H	-2.6974490000	0.2385580000	-0.2508540000
O	-3.7119660000	1.4261710000	0.2995760000
H	-3.9737250000	2.2116370000	-0.1925450000
H	-4.4339760000	1.2301140000	0.9066200000

4IV: E = -452.498491

Ag	-0.3261290000	-0.0720250000	0.0225790000
O	-2.5948630000	0.1257520000	0.0293240000
H	-3.1766020000	-0.3726520000	-0.5592370000
H	-3.1126820000	0.3253920000	0.8200460000
O	1.2384360000	1.6069440000	-0.9720260000
H	1.0043680000	2.4922670000	-1.2817230000
H	1.7942880000	1.2279160000	-1.6667670000
O	1.5062190000	0.1783720000	1.6195260000
H	1.9721040000	0.9882940000	1.3674980000
H	1.5048890000	0.1561170000	2.5856410000
O	1.3615340000	-1.5713100000	-0.7967530000
H	1.2611840000	-2.3997730000	-1.2838470000
H	1.9899040000	-1.7504470000	-0.0834100000

4V: E = -452.591087

O	2.5980490000	2.3089310000	0.1271600000
H	3.0429330000	2.7654010000	-0.5948880000
H	3.0022960000	2.6300950000	0.9398560000
Ag	-0.7933830000	-0.0030870000	-0.0376650000
O	2.6195960000	-2.2996730000	0.1241670000
H	3.0537160000	-2.5956630000	0.9310740000
H	3.0690510000	-2.7444500000	-0.6024260000
O	-2.9527850000	0.0473210000	0.1671910000
H	-3.4068330000	-0.2661170000	0.9576970000
H	-3.5433810000	-0.0965670000	-0.5811610000
O	1.2895730000	-0.0004350000	-0.3016430000
H	1.8212790000	-0.8056280000	-0.1083170000
H	1.8144890000	0.8088680000	-0.1065780000

4VI: E = -452.588789

O	-2.7502050000	-0.4178030000	0.8537520000
H	-3.1197820000	-0.9618660000	1.5556350000
H	-3.4977370000	0.0561670000	0.4277320000
O	-4.7644830000	0.9546130000	-0.3664220000
H	-5.0775920000	1.8035730000	-0.0370420000
H	-5.5011790000	0.5675200000	-0.8507240000
O	-0.6238780000	-1.2127850000	-0.3364530000
H	-0.8189520000	-1.6468250000	-1.1720900000
H	-1.4905830000	-0.9125050000	0.0891070000
Ag	1.1463540000	-0.0888600000	-0.0815290000
O	2.9649450000	1.0379690000	0.2794640000
H	3.2739180000	1.7316430000	-0.3146040000
H	3.7422260000	0.6427720000	0.6911180000

4VII: E = -452.576939

O	-0.2271750000	1.9689850000	-0.6815560000
H	-1.1210580000	1.6864940000	-0.9312880000
H	0.0739750000	2.6148660000	-1.3300790000
Ag	0.7824250000	0.0006670000	-0.0330620000
O	-0.6771280000	0.0028270000	2.0243320000
H	-0.6471960000	0.7733270000	2.6049690000
H	-0.6447960000	-0.7646590000	2.6088170000
O	-0.2226370000	-1.9715430000	-0.6765210000
H	0.0800160000	-2.6185820000	-1.3231870000
H	-1.1172230000	-1.6919630000	-0.9269740000
O	-2.3445670000	-0.0032810000	-0.5161910000
H	-3.2662110000	-0.0046890000	-0.8002580000

H	-2.3594290000	-0.0020240000	0.4513890000

5I: E = -529.052055

O	-2.2494040000	-0.8486570000	-0.1879410000
H	-2.5549880000	-1.7125470000	0.1029440000
H	-2.9785840000	-0.2034030000	-0.0348440000
O	3.8673740000	0.7081490000	-0.0100510000
H	4.4713820000	0.8062540000	-0.7559130000
H	4.3933710000	0.8903520000	0.7783200000
O	-4.1822120000	0.9919850000	0.1331290000
H	-4.6127100000	1.2363000000	0.9591540000
H	-4.8256030000	1.1539430000	-0.5651540000
Ag	-0.1321820000	-0.2599470000	-0.0291920000
O	1.1850540000	1.6579040000	0.0119110000
H	2.1484890000	1.5522680000	-0.0656450000
H	0.9521260000	2.5246470000	-0.3338150000
O	1.9235780000	-1.3706020000	0.2028070000
H	2.1258270000	-2.2879490000	-0.0037760000
H	2.7381240000	-0.8525990000	0.0919060000

5II: E = -529.055770

Ag	0.2926600000	-0.0097980000	-0.2184150000
H	3.0711220000	-0.0005650000	-0.1845720000
H	-3.4149250000	2.7785270000	0.9782700000
H	-2.2621710000	-0.7904630000	0.3341520000
H	4.8901150000	-0.6919430000	1.0923370000
H	-3.6217920000	-2.6214230000	-0.5056020000
H	4.6974540000	0.8211740000	1.3324660000
H	-3.6383580000	2.6123860000	-0.5368700000
H	-2.2545730000	0.8173060000	0.3214490000
H	2.6111910000	0.3048030000	-1.6684670000
H	-3.4414350000	-2.7367990000	1.0206150000
O	-3.1092040000	-2.2862570000	0.2371030000
O	2.3308090000	-0.0870560000	-0.8361780000
O	-1.7047980000	0.0117110000	0.4401580000
O	4.2713150000	0.0363620000	0.9723990000
O	-3.0870770000	2.3211740000	0.1967370000

5III: E = -529.049992

Ag	-0.7625140000	-0.1087190000	-0.0524960000
H	0.5986310000	2.5001400000	0.1435000000
H	3.1058760000	-3.2712780000	-0.4642390000
H	3.0220780000	2.5211530000	0.7864200000
H	2.9798990000	2.6054040000	-0.7480490000
H	-3.6439440000	-0.3087320000	0.2179860000
H	1.9827360000	0.3646380000	-0.1802440000
H	2.9246890000	-3.1202520000	1.0584090000
H	1.8934030000	-1.2210270000	-0.1217070000
H	-0.8648440000	3.0364850000	0.0782170000
H	-3.1670200000	-1.6990730000	-0.2691700000
O	-2.8703990000	-0.8765210000	0.1348810000
O	-0.3345830000	2.2530750000	0.2525450000
O	1.4167550000	-0.4001250000	-0.3670310000
O	2.5137100000	2.2433730000	0.0150620000
O	2.6503510000	-2.7570120000	0.2103140000

5IV: E = -529.053809

O	-5.2845710000	0.9760180000	0.0490980000
H	-5.5507640000	1.7979740000	0.4740680000
H	-6.0644900000	0.6473660000	-0.4098450000
O	2.3721850000	1.0944320000	-0.0664530000
H	3.2211490000	0.6776640000	0.2288290000
H	2.5792120000	1.8129390000	-0.6713860000
O	-3.2014260000	-0.5319800000	0.9774490000
H	-3.5220160000	-1.1217470000	1.6660680000
H	-3.9701180000	-0.0133410000	0.6567950000
Ag	0.5958380000	-0.0595500000	-0.2919480000
O	-1.1575510000	-1.2359340000	-0.4240240000
H	-1.9854630000	-0.9680570000	0.0832430000
H	-1.4224310000	-1.6190690000	-1.2651570000
O	4.5789190000	-0.0662000000	0.8350500000
H	5.2641340000	-0.5110990000	0.3253060000
H	4.9859630000	0.2055230000	1.6646790000

5V: E = -529.048051

Ag	-0.2297700000	-0.8274230000	0.0373340000
O	-2.2142230000	2.1002080000	-0.5578760000

H	-1.3568470000	2.3791260000	-0.2005770000
H	-2.8166970000	2.8481600000	-0.5017920000
O	0.3549600000	1.8321200000	0.6961110000
H	1.2724200000	1.9519710000	0.4052730000
H	0.3343630000	2.0960230000	1.6230920000
O	-2.3489530000	-0.4301000000	0.1459220000
H	-2.4954630000	0.5142460000	-0.1267530000
H	-2.9713000000	-0.9865890000	-0.3335170000
O	1.9035630000	-1.1723950000	-0.2588650000
H	2.3795560000	-1.9455920000	0.0598550000
H	2.4874060000	-0.3905990000	-0.1981020000
O	3.1400170000	1.3261590000	-0.2380800000
H	3.8802250000	1.5517580000	0.3389960000
H	3.4026220000	1.6224400000	-1.1188620000

5VI: E = -529.045835

Ag	-0.8542220000	-0.1349890000	-0.0706220000
O	-2.1942840000	1.6069620000	0.4056590000
H	-2.9972630000	1.4726140000	0.9224360000
H	-2.4151990000	2.2471280000	-0.2807330000
O	2.5758680000	2.5085070000	-0.6808340000
H	2.7156550000	3.4249350000	-0.4192540000
H	3.2660470000	2.3105650000	-1.3230550000
O	1.4346700000	0.5643660000	0.8753980000
H	1.8710760000	1.2799800000	0.3602520000
H	1.4445010000	0.8541710000	1.7943990000
O	-0.1024690000	-2.0489020000	-0.8214610000
H	-0.6546100000	-2.8369820000	-0.8224270000
H	0.7629440000	-2.2713890000	-0.3868410000
O	2.1463280000	-2.1750030000	0.5237520000
H	2.2634710000	-1.2306180000	0.7166350000
H	3.0108830000	-2.5533590000	0.3377230000

5VII: E = -529.048660

Ag	1.1483960000	-0.0006060000	0.0024700000
H	-3.8775540000	0.0044870000	-0.8497310000
H	1.3837780000	-2.6787290000	-0.5582890000
H	-2.3072820000	1.6791930000	0.0240290000
H	-2.1758430000	3.1975050000	0.3497140000
H	1.3872840000	2.6767800000	-0.5575090000
H	-0.0545530000	2.3438670000	0.0209170000
H	-2.3114600000	-1.6777770000	0.0195780000
H	-2.1802670000	-3.1954000000	0.3492850000
H	-0.0574250000	-2.3435970000	0.0204130000
H	-4.0243620000	0.0009470000	0.6776120000
O	0.9093340000	-2.1183210000	0.0647630000
O	-1.7104760000	2.4440300000	-0.0269170000
O	0.9119480000	2.1174670000	0.0658590000
O	-1.7133610000	-2.4419800000	-0.0254800000
O	-3.3670590000	0.0014540000	-0.0297370000

5VIII: E = -529.049291

O	-4.5515490000	1.4158180000	0.8098080000
H	-5.3839610000	0.9579730000	0.9655800000
H	-4.7151170000	2.3417010000	1.0172950000
Ag	1.2272950000	0.1790300000	-0.0405970000
O	-0.8721410000	-3.3145500000	0.4433600000
H	-0.8955590000	-4.0747850000	-0.1474790000
H	-1.3757510000	-3.5664260000	1.2242640000
O	-2.6140910000	0.6800770000	-1.0079180000
H	-3.3240950000	0.9272790000	-0.3807650000
H	-2.9936170000	0.7174730000	-1.8904840000
O	3.0427140000	1.2653580000	0.4490160000
H	3.3930610000	1.2971460000	1.3464570000
H	3.7946090000	1.2902930000	-0.1538850000
O	-0.5185880000	-0.8328390000	-0.5901730000
H	-1.3556500000	-0.3071480000	-0.7107440000
H	-0.7175420000	-1.7088210000	-0.1949020000

6I: E = -605.512357

O	2.7937310000	-0.6581010000	-0.5427280000
H	3.1886750000	-1.5272860000	-0.4293630000
H	3.4908660000	0.0132060000	-0.3608510000
O	4.6330020000	1.2656060000	-0.1199100000
H	5.1268910000	1.4266770000	0.6907890000
H	5.1825060000	1.5909430000	-0.8408240000
O	-1.1996160000	-1.6441250000	0.3391550000

H	-1.3889860000	-2.4879000000	-0.0809360000
H	-2.0420210000	-1.1594300000	0.4380110000
O	-5.2805520000	0.6380700000	-0.9687940000
H	-5.8986140000	-0.0345130000	-1.2730870000
H	-5.7013470000	1.4866770000	-1.1412330000
Ag	0.6848280000	-0.2876010000	0.0014610000
O	-3.2221030000	0.2149810000	0.7692210000
H	-3.9767090000	0.3689690000	0.1573840000
H	-3.5932350000	0.2085690000	1.6582820000
O	-0.7486570000	1.4439280000	0.5575210000
H	-0.6863450000	2.3477140000	0.2357690000
H	-1.6950440000	1.2007520000	0.6016640000

6II: E = -605.516416

Ag	0.0000070000	0.1227320000	0.0000030000
H	2.6591270000	0.6493300000	-0.3055810000
H	-3.7039480000	-2.0179570000	-2.2000240000
H	-2.6591170000	0.6493250000	0.3056030000
H	4.0851290000	2.4137680000	-1.0620110000
H	-3.9460910000	1.4254790000	2.2365310000
H	3.9460560000	1.4255990000	-2.2364980000
H	-3.6536930000	-2.7959380000	-0.8716450000
H	-2.5469580000	-0.6399000000	-0.6410040000
H	2.5469670000	-0.6399340000	0.6409920000
H	-4.0851700000	2.4136930000	1.0620830000
O	-3.5846710000	1.6526340000	1.3739020000
O	2.0819950000	0.1625980000	0.3209280000
O	-2.0819810000	0.1626250000	-0.3209280000
O	3.5846430000	1.6527110000	-1.3738540000
O	-3.2619480000	-2.0551160000	-1.3453270000
H	3.6536710000	-2.7959990000	0.8715670000
O	3.2619370000	-2.0551810000	1.3452650000
H	3.7039200000	-2.0180580000	2.1999720000

6III: E = -605.510092

O	2.5998110000	-0.6004560000	-0.0603370000
H	2.9100600000	0.3147250000	0.0767050000
H	3.2496090000	-1.2021520000	0.3146080000
O	3.0610460000	2.1562700000	0.1225000000
H	3.4711580000	2.5804300000	0.8854940000
H	3.5004150000	2.5309420000	-0.6505530000
O	-1.5981150000	-1.8565560000	-0.0321230000
H	-1.7851230000	-2.5935510000	0.5569880000
H	-2.3516260000	-1.2171640000	0.0322780000
O	-2.4834060000	2.6894710000	0.0041030000
H	-2.7108580000	3.2348180000	-0.7586580000
H	-2.7556510000	3.1988200000	0.7770420000
Ag	0.3800510000	-0.9121000000	-0.0144590000
O	-3.5183240000	-0.0164920000	0.1273930000
H	-4.3829260000	-0.1334420000	-0.2780130000
H	-3.2756360000	0.9184700000	0.0236510000
O	0.1805080000	1.5172000000	-0.1882590000
H	-0.6189530000	2.0504950000	-0.0608580000
H	0.9549830000	2.0707950000	-0.0253170000

6IV: E = -605.508461

Ag	0.0013050000	-1.0773750000	0.0002060000
H	2.8280750000	2.4302940000	-0.2182650000
H	-0.8477590000	1.7981040000	-1.3448320000
H	0.8434300000	1.8021260000	1.3438730000
H	-2.7521560000	-1.4765370000	0.3423790000
H	-2.4054000000	0.0207420000	-0.0346790000
H	0.0997800000	1.6295970000	2.7013340000
H	2.7558780000	-1.4704690000	-0.3404990000
H	2.4054590000	0.0263690000	0.0349880000
H	-0.1040290000	1.6249650000	-2.7021770000
H	1.4893810000	1.8776970000	-0.8367030000
H	-1.4937550000	1.8751280000	0.8353990000
H	-2.8337940000	2.4238320000	0.2163460000
O	-2.1580860000	-0.9218570000	-0.1738190000
O	2.1340000000	1.7728850000	-0.1024880000
O	0.0006760000	1.5299740000	1.7488320000
O	2.1602640000	-0.9166700000	0.1748640000
O	-2.1381430000	1.7679630000	0.1012940000
O	-0.0045150000	1.5270510000	-1.7495370000

6V: E = -605.510373

O	-1.5551610000	-1.1765000000	-0.0601850000
H	-1.7458390000	-2.0968800000	0.1398940000
H	-2.3007650000	-0.6224060000	0.3022140000
O	4.2703260000	1.2560250000	-0.2060390000
H	4.7584950000	1.5519160000	-0.9836280000
H	4.8538530000	1.4151870000	0.5458570000
O	-3.4663080000	0.3788450000	0.8331330000
H	-3.7170420000	0.4420520000	1.7590780000
H	-4.2813500000	0.5230360000	0.3097000000
Ag	0.4968690000	-0.4216280000	0.0018990000
O	1.4791970000	1.7118180000	0.2295850000
H	2.4268930000	1.8091720000	0.0415860000
H	1.0560090000	2.5591430000	0.0626800000
O	2.7142220000	-1.1257060000	-0.1012940000
H	3.0326840000	-1.9468900000	-0.4875710000
H	3.4042520000	-0.4513050000	-0.2206880000
O	-5.7346790000	0.8033940000	-0.6661840000
H	-6.0484130000	1.6657600000	-0.9560930000
H	-6.4523660000	0.1847390000	-0.8343980000

6VI: E = -605.509599

Ag	1.1477270000	-0.2486920000	-0.0792360000
O	-2.6558860000	-0.2591480000	0.8507420000
H	-3.3950270000	-0.4656180000	0.2361800000
H	-3.0670660000	-0.0324900000	1.6914650000
O	-4.7114380000	-0.8488520000	-0.8454700000
H	-5.1010420000	-1.7172450000	-0.9885240000
H	-5.1702620000	-0.2444510000	-1.4374120000
O	-0.0984700000	3.8822900000	0.1649230000
H	0.0049970000	4.3884020000	0.9769810000
H	-0.2885420000	4.5224060000	-0.5285770000
O	-0.5151730000	1.2010140000	-0.2209320000
H	-1.3463370000	0.8845240000	0.1788290000
H	-0.4059470000	2.1551100000	-0.0339910000
O	3.2731030000	-0.9734030000	-0.3089660000
H	3.9591980000	-0.4112640000	-0.6851270000
H	3.4645720000	-1.8733490000	-0.5949300000
O	-0.4186590000	-1.9251370000	0.6966450000
H	-0.2988250000	-2.6213120000	1.3492470000
H	-1.2867090000	-1.5103010000	0.8644460000

6VII: E = -605.509638

O	-2.6608680000	0.3782330000	0.7090830000
H	-3.5388420000	0.1690580000	0.3746370000
H	-2.4742970000	1.3389820000	0.5306690000
O	-1.7689830000	2.7632130000	0.0770410000
H	0.1702980000	1.5223180000	-2.1709330000
H	-1.7407030000	3.5902220000	0.5670390000
O	0.8780050000	-1.8705910000	-0.3428690000
H	1.1302890000	-2.6493950000	0.1627820000
H	1.6723640000	-1.2978270000	-0.4599770000
O	4.4489130000	0.8181580000	1.1648420000
H	5.0689390000	0.2633750000	1.6497820000
H	4.7573460000	1.7238140000	1.2746890000
Ag	-0.9413450000	-0.7696180000	0.0999980000
O	2.7951490000	-0.0156150000	-0.8179270000
H	3.4125030000	0.2866290000	-0.1110630000
H	3.3414250000	-0.1806220000	-1.5946640000
O	0.3177530000	1.3892250000	-1.2279450000
H	-0.8832740000	2.5914790000	-0.2833790000
H	1.2473960000	1.1130270000	-1.1373150000

6VIII: E = -605.507359

Ag	-1.0920890000	0.2311700000	-0.0477570000
H	3.6108320000	-0.8391160000	0.2802110000
H	-0.9588390000	2.9288600000	-0.6413560000
H	1.6880560000	-2.0906740000	-0.6467260000
H	1.3124620000	-3.5500600000	-1.0710760000
H	-2.1441060000	-2.2780080000	-0.4563900000
H	-0.5589260000	-2.3262420000	-0.3669100000
H	0.7264210000	-0.6407500000	2.8613650000
H	1.0723130000	0.8383830000	2.6253630000
H	2.4252810000	1.0527440000	-0.7576300000
H	2.5905570000	2.3335620000	-1.6442550000
H	0.4914910000	2.2862900000	-0.5354340000
H	2.2472160000	-0.4711110000	0.9398280000
O	-0.4331830000	2.2743960000	-0.1700730000
O	0.9547030000	-2.6691670000	-0.9263290000

O	-1.3826880000	-1.8976120000	-0.0061060000
O	0.9679260000	-0.0081180000	2.1744800000
O	2.0596730000	1.9354300000	-0.9484080000
O	2.6867490000	-0.6485390000	0.0836350000

6IX: E = -605.498503

O	1.4116470000	0.4002880000	-1.3592280000
H	2.1854500000	0.4102880000	-0.7666300000
H	1.0523780000	1.3007680000	-1.3641150000
O	0.6462410000	-0.4116410000	1.8331040000
H	0.1073420000	0.3299920000	2.1407800000
H	1.5654260000	-0.1094320000	1.8106040000
O	-1.6426390000	1.5401200000	1.4434720000
H	-2.4714430000	-0.0361500000	0.7132520000
H	-2.1515960000	2.1064010000	2.0339980000
Ag	-0.2260340000	-0.8722510000	-0.4125180000
O	-0.3890220000	2.7934420000	-0.9280890000
H	-0.9957230000	2.8760210000	-1.6747180000
H	-0.0035420000	3.6721130000	-0.8235790000
O	-2.4370490000	-0.7858430000	0.0899740000
H	-2.9229540000	-1.5211970000	0.4781490000
H	-1.3199410000	2.1102830000	0.7245050000
O	3.3714940000	0.1677520000	0.7275480000
H	3.8973530000	-0.6410180000	0.6994620000
H	3.9954690000	0.8647880000	0.9623790000
